# Self‐disgust and the body as a target in self‐harm and suicidal behavior: A narrative review

**DOI:** 10.1002/pcn5.70443

**Published:** 2026-11-10

**Authors:** Dorin Jelaga, Mihaela Belous

**Affiliations:** ^1^ Department of Mental Health, Medical Psychology and Psychotherapy “Alexandru Nacu”, and Mental Health Laboratory Nicolae Testemitanu State University of Medicine and Pharmacy Chisinau Republic of Moldova

**Keywords:** non‐suicidal self‐injury, self‐conscious emotions, self‐disgust, self‐harm, suicidal behavior

## Abstract

Self‐harm and suicidal behavior are commonly framed as failures of emotion regulation, yet the emotions that make the body the target of harm remain under‐specified. This narrative review synthesizes evidence on self‐disgust as a candidate mechanism in non‐suicidal self‐injury (NSSI) and in suicidal ideation and behavior. Self‐disgust is an aversive self‐directed emotion organized around the appraisal of the self, and often the body, as revolting or contaminated. Cross‐sectional studies consistently link self‐disgust with NSSI, suicidal ideation, and suicide‐risk scores, and qualitative accounts place it at the center of some suicidal crises. Longitudinal studies from China suggest it mediates reciprocal links between emotional abuse and suicidal ideation and a path from body‐image concerns to NSSI. Two prospective studies testing different roles did not support them, and the only induction study of self‐harm urges is equivocal. In some studies, self‐disgust explains variance beyond depression and constructs of the interpersonal theory of suicide. Its boundaries with shame and self‐criticism are contested, and, across disorders, associations shrink when other negative self‐referential emotions are controlled for. The central proposal, that the contamination‐and‐elimination logic of disgust explains why distress is enacted on the body, builds on earlier suggestions and remains an untested hypothesis. On this hypothesis, self‐disgust is an embodied emotion whose action tendency of cleansing and removal may correspond to the phenomenology of cutting and, in some people, to a wish to eliminate a self experienced as contaminated. Implications for assessment and intervention, which remain untested, are outlined with falsifiable predictions for research.

## INTRODUCTION

Theories of self‐harm have long emphasized emotion regulation. Non‐suicidal self‐injury (NSSI) is widely understood as a way of managing intolerable internal states, and affect regulation is its best‐supported function.^1^, ^2^ Suicidal behavior, too, is explained in many theoretical accounts by emotional pain.^3^ In this review, self‐harm is used as an umbrella term for NSSI and suicidal acts, and suicidality refers to suicidal ideation and behavior together. NSSI and suicidal acts are distinguished wherever the evidence allows. The emotion‐regulation account holds that people harm themselves to change how they feel, but it says little about why the body is so often the site and the instrument of that regulation. Why is distress discharged by cutting, burning, or otherwise damaging the physical self, and not only by withdrawal, rumination, or reassurance‐seeking? Existing answers are mainly pragmatic or functional. Self‐injury may be chosen because it is fast and easily accessible,^4^ or because, in some people, the natural barriers against hurting oneself are weakened, so that its benefits come within reach.^5^ These accounts explain when self‐injury pays off, but not which emotions carry a bodily action tendency, meaning a readiness to act on the body. The question calls for attention to particular emotions and not to negative affect as a whole.

Research on self‐conscious emotions and self‐evaluation in relation to self‐harm has concentrated on shame and self‐criticism.^6^, ^7^, ^8^ Neither construct ignores the body. Shame can concern the body, as in body shame,^7^ and the hated‐self form of self‐criticism includes a wish to hurt oneself.^6^ Yet shame chiefly motivates hiding and social withdrawal, and self‐criticism motivates correction or punishment of the self. Neither explains why harm should take the specific form of cleansing or removal. Disgust is different, because as a basic emotion it is organized around contamination.^9^ Its rejection responses serve to keep a contaminant outside the body or to stop it from spreading within the body.^10^ When disgust is turned on the self (self‐disgust), the self, and frequently the body, becomes the contaminated object.^11^, ^12^, ^13^ This makes self‐disgust a theoretically distinctive candidate for explaining the bodily form of self‐harm, and yet it has received less attention than shame in this context.

The construct of self‐disgust now has its own literature, validated measures such as the Self‐Disgust Scale^12^ and later instruments tested in clinical populations,^14^ and one systematic review of its clinical utility across disorders.^15^ That review concluded that self‐disgust is strongly related to depression, trauma‐related distress, and body‐image difficulties. It also noted two limits that apply throughout the present review. Across disorders, associations shrink when other negative self‐referential emotions are controlled for, and causal claims are constrained by the largely cross‐sectional evidence. That review covered self‐harm in a short subsection based on three studies, none of them longitudinal, and from these it tentatively suggested that self‐disgust and self‐harm urges may influence each other. It discussed suicidality only within sections on other difficulties, and it predates most of the longitudinal studies now available. Other work has treated parts of the topic. A conceptual article proposed a role for disgust with life, including disgust with oneself, in severe suicidal ideation and behavior.^16^ A review in Chinese argued that self‐disgust is a key factor in suicidal ideation and that disgust may lead to attacks on oneself and to suicide, much as autoimmunity turns the body against itself.^17^ A book chapter described how self‐disgust may appear in the experience of self‐harm,^10^ and a systematic review examined humiliation and shame, which are neighboring but distinct constructs, in relation to self‐harm and suicidality.^7^ To our knowledge, no review has yet synthesized the evidence on self‐disgust for NSSI and suicidality together, including the prospective studies published since 2024.

This review has three aims. The first is to define self‐disgust and to situate it against shame, guilt, and self‐criticism, treating the contested boundaries, especially with self‐criticism, as a substantive question instead of assuming distinctness. The second is to synthesize the evidence linking self‐disgust to NSSI and to suicidal ideation and behavior, and to trace the developmental antecedents of self‐disgust. The third, and the review's principal contribution, is to develop the hypothesis that the contamination‐and‐elimination logic of disgust, by which what feels contaminated is cleansed or removed, explains why the body becomes the target. Parts of this idea have precursors, which are credited below, so the main additions are its extension to the bodily forms of NSSI and a set of tests. That hypothesis is then examined critically. Throughout, convergent correlational evidence is treated as suggestive of mechanism rather than as proof of it.

This review is narrative rather than systematic. Studies were identified in September 2026 by searching PubMed, Europe PMC, and Crossref for self‐disgust and related terms (self‐directed disgust, disgust with oneself or with life, and self‐loathing) combined with terms for self‐harm, NSSI, and suicidal ideation and behavior. The search strings differed between databases and are given in full in Supporting Information S1, with the number of records returned by each PubMed and Europe PMC string. A further search covered the construct and measurement literature, and a few studies were found in the reference lists of related articles. Peer‐reviewed articles and book chapters were eligible, and unpublished theses were not included. Publications had to be in English or to have an English abstract, and Chinese‐language databases were not searched. Records were screened on title and abstract. DJ directed the searches and the data extraction and made all eligibility decisions, and MB reviewed the synthesis and the manuscript. Screening and data extraction were not duplicated, so there were no disagreements between reviewers to resolve. Table 2 lists the studies identified by these searches that tested self‐disgust as a construct in relation to a self‐harm or suicidality outcome in individuals. Other studies were included when they informed the construct, its measurement, or the proposed mechanism.

## WHAT SELF‐DISGUST IS, AND WHAT IT IS NOT

Self‐disgust denotes an aversive, often enduring orientation toward the self in which aspects of one's person or body are experienced as revolting, repellent, or contaminated. Overton et al.^12^ originally distinguished two facets, a disgusting self (revulsion at enduring aspects of oneself that do not depend on context) and disgusting ways (revulsion at one's own behavior), and found a statistical indirect association from dysfunctional cognitions to depressive symptoms through self‐disgust. That finding suggested that self‐disgust is more than a by‐product of low mood. Qualitative studies synthesized in the review by Clarke et al.^15^ describe the emotion as a visceral, nausea‐like reaction to oneself, together with social withdrawal and efforts to cleanse or suppress parts of the self. Measurement has developed alongside these accounts. The Self‐Disgust Scale was followed by a revised version with separate physical and behavioral subscales, used for example by Burden et al.^18^ Another instrument, the Questionnaire for the Assessment of Self‐Disgust, has been used in patients with various mental disorders^11^ and tested psychometrically in clinical samples.^14^ These instruments divide the object of disgust in different ways. Physical self‐disgust concerns one's appearance and body, disgust at the overall self concerns enduring features of the person, and behavioral self‐disgust concerns one's actions. The Questionnaire for the Assessment of Self‐Disgust combines appearance and personality in a single personal subscale, separate from behavior,^11^ whereas the revised Self‐Disgust Scale separates the physical from the behavioral self. The distinction matters here, because only physical self‐disgust is directed at the body as such. A broader disgust‐with‐life scale covers disgust with oneself, with others, and with the world,^19^, ^20^, ^21^ and multidimensional variants have been used in recent research on suicidal ideation.^22^ Whether self‐disgust is best modeled as an implicit or an explicit emotional schema is itself an active question.^23^

Distinguishing self‐disgust from its neighbors is essential, because the review's claims depend on it (Table 1). Relative to shame, self‐disgust is theorized to rest on contamination‐based appraisals and to be experienced more viscerally.^15^ It is also defined as disgust directed at core and stable features of the self, which often include the body.^13^, ^15^ Not all authors separate the two emotions. One study reviewed here treats self‐disgust as theoretically identical to shame,^27^ men interviewed after a suicide attempt often found the two hard to tell apart,^3^ and people who self‐injure describe shame itself as including a sense of being contaminated.^26^ Experimental work that confronted patients with their own faces nonetheless measured shame and self‐disgust as separate self‐conscious emotions and found both heightened in borderline personality disorder.^24^ Relative to guilt, which concerns specific transgressions and motivates reparation, self‐disgust concerns the self as an object and motivates removal rather than repair. Relative to low self‐esteem, self‐disgust is an emotion with an action tendency and not merely a negative self‐evaluation.

**Table 1:** Self‐disgust distinguished from adjacent self‐conscious and self‐evaluative constructs.

Construct	Core appraisal	Prototypical action tendency	Primary target	Representative sources
Self‐disgust	The self, and often the body, is contaminated or revolting	Expel, cleanse, remove, distance	Self and body	Overton et al.^12^ and Powell et al.^13^
Shame	The self is defective and exposed to others	Hide, withdraw, conceal	The socially evaluated self	Biermann et al.,^24^ Sadath et al.,^7^ and Sheehy et al.^8^
Guilt	A specific act was wrong	Repair, make amends	One's behavior	Sheehy et al.^8^
Self‐criticism	The self is inadequate (an evaluative stance)	Correct or punish the self	The self as appraised in thought	Gilbert et al.^6^
Low self‐esteem	The self is of low worth overall	No specific action tendency	The self as a whole	–

*Note*: The distinctions are theoretical. Empirical discriminant validity is partial and contested, especially for the boundary between self‐disgust and self‐criticism (see text). The hated‐self form of self‐criticism overlaps substantially with self‐disgust,^6^, ^25^ and feelings of contamination can also accompany shame.^26^ Representative sources are works in this review that define or measure the construct in relation to self‐disgust or self‐harm. Low self‐esteem is included for conceptual contrast only, so no source is listed.

The most demanding boundary is with self‐criticism, and here the literature is divided. Gilbert et al.^6^ identified a hated‐self form of self‐criticism, marked by the wish to hurt oneself alongside self‐directed disgust and hatred. This form overlaps substantially with the self‐disgust construct, and at least one self‐harm study explicitly treats self‐disgust as a component of self‐criticism.^25^ The two are clearly related, but self‐disgust may be an emotional core that can be separated from the cognitive operations of self‐critical thought. Its disgust‐specific action tendency of expulsion and cleansing may be what carries incremental explanatory value. Quantitatively, self‐disgust also overlaps with depression and anxiety, with pooled correlations of 0.52 (95% CI [0.45, 0.58]) and 0.45 (95% CI [0.40, 0.51]), respectively, in a three‐level meta‐analysis,^28^ and the emotion appears across diagnoses.^11^ These confidence limits were converted to correlations from the Fisher *z* values reported in that meta‐analysis. In summary, self‐disgust is a distinguishable but overlapping member of a family of aversive self‐conscious states. Its value for the self‐harm field rests on whether it adds explanatory power once its neighbors are accounted for, a question this review returns to repeatedly.

## SELF‐DISGUST AND NON‐SUICIDAL SELF‐INJURY

The cross‐sectional association between self‐disgust and NSSI is one of the more consistent findings in this literature, and the evidence has shifted in recent years toward longitudinal designs (Figure 1 and Table 2). Early cross‐sectional work established the correlation and proposed mediation. In a student sample, a statistical indirect association was observed from depressive symptoms to NSSI through self‐disgust, with no remaining significant direct association, and a partial indirect association from sexual abuse to NSSI.^25^ In the same study, participants with recent NSSI reported higher self‐disgust than those with past NSSI. Later cross‐sectional studies extended this pattern of indirect associations to other adverse experiences. Emotional neglect was statistically linked to NSSI through three parallel pathways, one of which ran through high self‐disgust and then emotion regulation difficulties.^33^ Parental psychological control was statistically linked to adolescent NSSI through self‐disgust and low resilience.^34^ In cross‐sectional designs, such mediation is a statistical indirect effect and does not establish a sequence in time.

**Figure 1:**
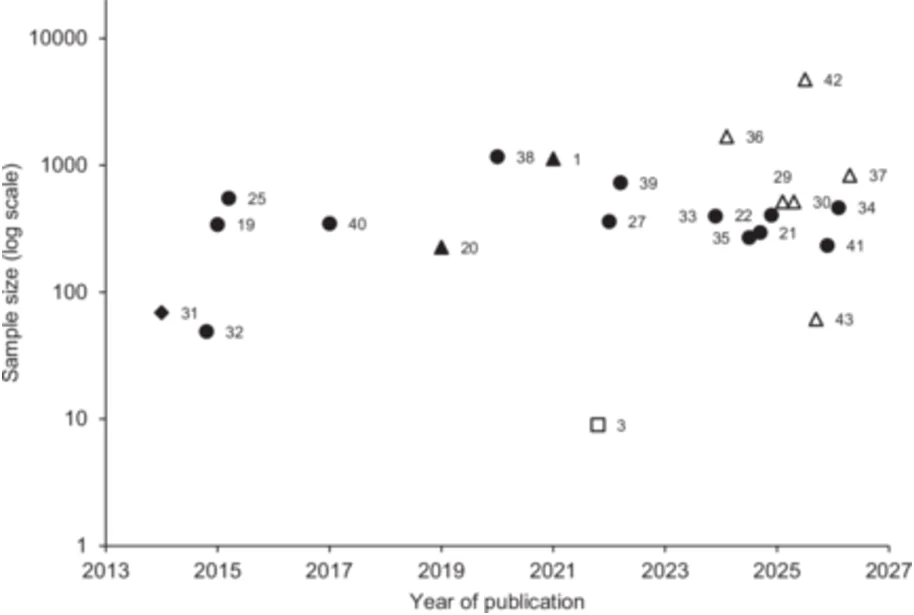
Studies relating self‐disgust to self‐harm and suicidality, by year of publication, sample size, and design. Each marker represents one of the 23 studies in Table 2, plotted by year of publication and by sample size on a logarithmic scale, and is labeled with the study's reference number. Filled circles show crosssectional studies; open triangles indicate longitudinal studies that supported the tested role for self‐disgust, whereas filled triangles show longitudinal studies that did not; the filled diamond represents the quasi‐experimental study; and the open square represents the qualitative study. Studies published in the same year are spread around that year in horizontal steps of 0.2 years, in order of sample size, to reduce overlap. Every marker stays within half a year of its year of publication, and its position within that range carries no information. Ying et al.^29^ and Zhang et al.,^30^ which appear to share a sample, are shown side by side. Six of the eight longitudinal studies, all from China, were published from 2024 onwards and include the two largest samples. The two earlier longitudinal studies tested different roles and did not support them, one as a moderator over 4‐month intervals^1^ and one as a mediator in a relatively low‐risk student sample over 1‐month intervals.^20^ The smallest samples come from the qualitative study, the scar study, the daily diary study (plotted as longitudinal), and the quasi‐experimental study.

**Table 2:** Studies relating self‐disgust to self‐harm and suicidality.

Study, country	Design and timing	Sample	Self‐disgust measure	Outcome, measure	Main finding and key estimate	Covariates adjusted	Analytic model
Smith et al.^25^†, NR	Cross‐sectional	549 college students	NR	NSSI status (recent, past, none), measure NR	Statistical indirect association of depressive symptoms with NSSI via self‐disgust, with no remaining significant direct association, and a partial one for sexual abuse. Higher in recent than in past self‐injurers. Estimate NR	NR	Mediation analysis
Abdul‐Hamid et al.,^31^ UK	Quasi‐experimental pilot	17 with BPD, 27 with MDD, 25 controls (all women)	Disgust ratings on visual analog scales and disgust labels in written narratives	Self‐harm urge, self‐rated change on a visual analog scale	Body‐focused disgust higher in BPD. Narrative disgust labels, but not analog ratings, associated with urge change. Narrative labels and urge change, *p* = .009 (person task) and .002 (body task). Analog ratings not significant (one subgroup exception)	None	Non‐parametric group comparisons, Fisher's exact tests
Bachtelle and Pepper^32^†, NR	Cross‐sectional	49 college students with NSSI scars	NR	Scar meaning and likelihood of future NSSI, measure NR	Correlated positively with scar‐related shame and negatively with scar‐related growth. Estimate NR	NR	Correlations
Erol and Inozu^33^†, NR	Cross‐sectional	397 university students aged 18 to 30	NR	NSSI, measure NR	Statistical indirect association of emotional neglect with NSSI via self‐disgust and emotion regulation difficulties, alongside low distress tolerance and low self‐compassion. Estimate NR	NR	Path analysis
Jiang et al.,^34^ China	Cross‐sectional	463 adolescents	Chinese SDS (total)	NSSI, Adolescent NSSI Assessment Questionnaire (past 12 months)	Statistical indirect association of parental psychological control with NSSI via self‐disgust, together with resilience. *r* = .48. Adjusted *OR* = 2.73 [1.78, 4.18]. Indirect effect 0.45 [0.28, 0.69]	Gender, grade, family type, only‐child status	Logistic regression, serial mediation (PROCESS model 6)
Mohammadpour et al.,^35^ Iran	Cross‐sectional	269 adults with self‐harm in the past 6 months (89% female)	SDS (total)	Deliberate self‐harm, DSHI	Small statistical indirect association of adverse childhood experiences with self‐harm via self‐disgust, alongside shame. *r* = .20. Direct path not significant. Indirect effect 0.06 [0.011, 0.130]	None	Structural equation model with shame as parallel mediator
Ying et al.^29^†, China	Four‐wave longitudinal (6‐month intervals)	515 adolescents (50.7% boys)	NR	NSSI, measure NR	Within‐person, appearance concerns predicted NSSI over time through self‐disgust, reciprocally for general appearance concern. Estimate NR	NR	RI‐CLPM
Zhang et al.^30^†, China	Four‐wave longitudinal (6‐month intervals)	515 secondary school students (50.7% boys)	NR	NSSI and SI, measure NR	Within‐person, reciprocal links of FNE and NSSI through self‐disgust, and a one‐way link from FNE to SI through self‐disgust. Estimate NR	NR	RI‐CLPM
Shen et al.^36^†, China	Three‐wave longitudinal (6‐month intervals)	1684 adolescents (52.3% female)	NR	NSSI, measure NR	Mediated the link from problematic mobile social‐media use to NSSI. Estimate NR	NR	Latent growth curve and structural equation model
Hong et al.^37^†, China	Two‐wave longitudinal (6 months)	832 adolescents	NR	NSSI, measure NR	Predicted NSSI 6 months later, with a chain through depression and psychache. Estimate NR	NR	Structural equation model (chain mediation)
Hamza et al.,^1^ Canada	Three‐wave longitudinal (4‐month intervals)	1125 first‐year university students (72% female)	SDS, disgusting‐self subscale (at Time 1 only)	NSSI, adapted ISAS (past 4 months, yes or no)	Higher in students with NSSI, but did not moderate the path from stress to NSSI via emotional reactivity. *r* = .29 with NSSI at Time 1, .23 at later waves. Moderation not significant, χ^2^(6) = 8.80, *p* = .19	Age, gender	Autoregressive cross‐lagged path model, multi‐group comparison
Chu et al.^19^†, NR	Cross‐sectional	341 young adults (66% women)	Disgust with the self, others, and the world (measure NR)	SI, measure NR	Eating‐disorder symptoms and body dissatisfaction went with more SI only at high disgust with the self and the world. Estimate NR	NR	Moderation (multivariate regression)
Schienle et al.^38^†, NR	Cross‐sectional online survey	1167 participants (self‐selected)	NR	Lifetime suicidal ideation and behavior (suicide risk), measure NR	Strongest correlate of suicide risk among the variables assessed, with links through evasive coping. Estimate NR	Disgust proneness, coping styles, diagnoses of mental disorders	Hierarchical regression and mediation analyses
Akram et al.,^39^ UK and online	Cross‐sectional	728 participants (89% female)	SDS (total)	Suicidal thoughts and behaviors (composite), SBQ‐R total	Explained variance beyond body‐image disturbance, with a statistical indirect association of body‐image disturbance with the outcome. *r* = .50. β = .42 after body‐image disturbance. Indirect effect 1.01 [0.78, 1.25]	Age, sex, body‐image disturbance	Hierarchical regression, bootstrap mediation
Robison et al.,^21^ NR (online)	Cross‐sectional (Study 2)	295 sexual‐minority adults	DWLS, self‐disgust factor	SI, BSS (past week, attempt items excluded)	Related to SI over and above self‐blame and interpersonal‐theory constructs. *r* = .53. β = .21, *p* = .029, with all covariates	Self‐blame, burdensomeness, belongingness, homophobia measures, societal disgust	Hierarchical linear regression
Brake et al.^40^†, NR	Cross‐sectional	347 trauma‐exposed undergraduates	Disgusting self and disgusting ways facets	Suicide risk, measure NR	Statistical indirect association of PTSD symptoms with suicide risk via the disgusting self, not disgusting ways. Estimate NR	Depressive symptoms	Process model (mediation)
Gang et al.^41^†, South Korea	Cross‐sectional	233 community‐dwelling young women	NR	Suicidal behavior, measure NR	Associated with suicidal behavior alongside PTSD and impulsivity. β = 0.34, *p* < .001 (model *R* ^2^ = 44.6%)	PTSD, impulsivity	Multiple regression
Amraei et al.,^22^ Iran	Cross‐sectional	404 medical students (63% female)	MSDS (total)	SI, BSSI	One of three variables carrying statistical indirect associations of psychological distress with SI. *r* = .46. β = .30. Indirect effect 0.096 [0.06, 0.13]	None (perfectionism and emotion regulation difficulties as parallel mediators)	Path analysis
Mann et al.,^27^ USA	Cross‐sectional	360 adults recruited online (59% female)	SDS (total)	SI severity (labeled suicide risk by the authors), DSI‐SS (past 2 weeks)	Associated with SI severity, more strongly in sexual‐minority adults. *r* = .43. Slope *b* = 0.125 [0.085, 0.165] in sexual‐minority versus 0.033 [0.019, 0.046] in heterosexual adults	Age, income, education, emotional vulnerability	Hierarchical regression with sexual‐minority status as moderator
Gong and Zhou^42^†, China	Four‐wave longitudinal (6‐month intervals)	4731 early adolescents (44.9% girls)	NR	SI, measure NR	Within‐person, emotional abuse and SI predicted each other indirectly through self‐disgust. Estimate NR	NR	RI‐CLPM
Hom et al.,^20^ USA	Three‐wave longitudinal (baseline, 1 and 2 months)	226 undergraduates (89% female)	DWLS, disgust‐with‐self subscale	SI, DSI‐SS (past 2 weeks)	Statistical indirect association of insomnia with SI via disgust with self at baseline only. Prospectively, only disgust with others and the world mediated. Cross‐sectional indirect effect [.006, .027]. Longitudinal indirect effect not significant	Baseline mediator and baseline SI (longitudinal models)	Bootstrap mediation (PROCESS model 4)
Wang et al.,^43^ China	7‐day daily diary	61 college students	Three SDS items adapted to the day	Same‐day SI, single daily item adapted from the SBQ‐R	Same‐day indirect association of stress with SI via self‐disgust, at the within‐person level only. Within‐person *B* = 0.40 [0.23, 0.58]. Indirect effect *B* = 0.23 [0.12, 0.34]. Between‐person path not significant	None (demographics in sensitivity analyses)	Two‐level structural equation model
Mason et al.,^3^ UK	Qualitative grounded theory	9 men with a history of suicide attempt	Interviews (revised SDS used descriptively)	Suicide attempt, interview	Important emotion, central for 4 of 9, with distancing and fear of exposure	–	Constructivist grounded theory

*Note*: The table lists the studies identified by the searches described in the Introduction that tested self‐disgust in relation to a self‐harm or suicidality outcome in individuals. Studies of aggregate social‐media data,^44^ of disgust that was not directed at the self,^45^ and of the adjacent construct of self‐hate^46^ are therefore not listed. † The full text was not accessible to the authors, and the values come from the published abstract. Values in square brackets are 95% confidence intervals. In cross‐sectional studies, an indirect association is statistical and does not demonstrate a sequence in time. Ying et al.^29^ and Zhang et al.^30^ appear to draw on the same adolescent cohort. Supporting Information S2 gives an informal appraisal of each study.

Abbreviations: –, not applicable; BPD, borderline personality disorder; BSS, Beck Scale for Suicide Ideation; BSSI, Beck Scale for Suicidal Ideation; DSHI, Deliberate Self‐Harm Inventory; DSI‐SS, Depressive Symptom Inventory‐Suicidality Subscale; DWLS, Disgust With Life Scale; FNE, fear of negative evaluation; ISAS, Inventory of Statements About Self‐injury; MDD, major depressive disorder; MSDS, Multidimensional Self‐Disgust Scale; NR, not reported in the source used; NSSI, non‐suicidal self‐injury; OR, odds ratio; PTSD, posttraumatic stress disorder; RI‐CLPM, random‐intercept cross‐lagged panel model; SBQ‐R, Suicidal Behaviors Questionnaire‐Revised; SDS, Self‐Disgust Scale; SI, suicidal ideation.

More persuasive is the recent series of multi‐wave longitudinal studies, several of which use random‐intercept cross‐lagged panel models. These models separate stable differences between people (between‐person effects) from change within a person over time (within‐person effects). In a four‐wave study of adolescents, self‐disgust mediated the path from negative body image to NSSI. The temporal direction ran from concerns about fatness, shortness, and facial appearance to self‐disgust and then to subsequent NSSI, whereas general‐appearance concern and NSSI were reciprocally related through self‐disgust.^29^ A companion four‐wave study found that self‐disgust mediated reciprocal relations between fear of negative evaluation and NSSI.^30^ A three‐wave study used a latent growth curve model, which tracks the starting level of NSSI and its change over time. Problematic mobile social‐media use was linked to a higher initial level of NSSI, and self‐disgust mediated the association between problematic use and NSSI.^36^ A two‐wave study found that self‐disgust predicted NSSI 6 months later, and that a chain of depression and psychache, meaning intense psychological pain, carried at least part of this link.^37^ The model placed self‐disgust before depression by specification, so the order of the two was assumed rather than tested. Even so, the result cautions against reading every self‐disgust effect as a direct one.

Not every prospective test gives self‐disgust a special role. In a three‐wave study of 1125 university students, those who reported NSSI had higher self‐disgust at baseline, but high self‐disgust did not strengthen the path from stressful experiences to NSSI through emotional reactivity.^1^ The prospective studies differ in what they tested, so their results are neither direct replications nor failures to replicate. Hamza et al.^1^ measured the 'disgustingself' facet once and tested it as a moderator over 4‐month intervals. In contrast, Hom et al.^20^ (described below) examined disgust with self usinga disgust‐with‐life scale, testingit as a mediator in arelatively low‐risk student sample over 1‐month intervals. The supportive studies tested self‐disgust as a mediator or predictor in adolescents over 6‐month intervals, three of them with random‐intercept cross‐lagged panel models, or as a daily state in college students. These supportive studies also share features that limit their weight. All six supportive longitudinal or diary studies reviewed here, including those on suicidal ideation described below, come from China. Three share a senior author, and two appear to draw on the same or an overlapping adolescent cohort, so the six studies represent at most five independent samples. The apparent convergence therefore partly reflects shared samples and settings, and the generalizability of these findings remains uncertain.

One quasi‐experimental pilot study used a self‐relevant induction task with women who had borderline personality disorder or depression and with healthy controls. It used two disgust measures, emotion labels coded from participants’ written narratives and ratings on visual analog scales. Body‐focused disgust ratings were higher in the group with borderline personality disorder than in both comparison groups, and disgust labels in the narratives were associated with changes in self‐harm urges across the whole sample. The analog‐scale disgust ratings, however, were not significantly associated with urge change in the whole sample or in the group with borderline personality disorder, with one exception in the depression group.^31^ The design had no non‐self‐relevant control task, the groups were small, and participants were not selected for a history of self‐harm. To our knowledge, this is the only induction study of self‐disgust and self‐harm urges. Its null result on the quantitative index is therefore inconclusive, and the absence of adequately powered experiments is the main brake on strong mechanistic claims.

A questionnaire study of 49 students with NSSI scars found that scar‐related shame was associated with a higher self‐rated likelihood of future NSSI and with greater self‐disgust. Scar‐related growth, meaning a sense of growth found in one's scars, was in contrast associated with lower self‐disgust and a lower self‐rated likelihood of future NSSI.^32^ The authors suggested that people who find shame or guilt in their scars may form a more clinically severe group, and self‐disgust was part of that profile.

## SELF‐DISGUST AND SUICIDAL IDEATION AND BEHAVIOR

Evidence for a link with suicidality is broad in method and, once again, strengthening in design (Table 2). The outcomes differ, from suicidal ideation to composite suicide‐risk scores, lifetime suicidal behavior, and suicide attempts, and Table 2 gives the measure used in each study where the source reports it. Findings for ideation do not necessarily extend to suicidal behavior. The idea that disgust with oneself and with life contributes to suicidal ideation and behavior was set out early in conceptual work.^16^ In a large internet survey, self‐disgust emerged as the strongest correlate of suicide risk among the variables assessed, which included general disgust proneness, the trait tendency to feel disgust. It was also associated with reduced support‐seeking and with greater evasive coping.^38^ The incremental‐validity question, whether self‐disgust matters beyond established predictors, has been addressed directly, and the results are mostly supportive. Where correlations with the outcome were reported, they ranged from 0.20 to 0.53 (Table 2). Few studies adjusted for depression, shame, or self‐criticism. An indirect association through the disgusting‐self facet remained after depressive symptoms were controlled,^40^ and when shame was modeled alongside self‐disgust, the indirect association through self‐disgust was small.^35^ In one study, self‐disgust was related to suicidal thoughts and behaviors, measured as a composite score, beyond the variance explained by body‐image disturbance, and a statistical indirect association was observed from body‐image disturbance to these outcomes through self‐disgust.^39^ In a sexual‐minority sample, self‐disgust was associated with suicidal ideation beyond self‐blame and beyond perceived burdensomeness and thwarted belongingness, two constructs of the interpersonal theory of suicide. Its correlation with ideation was 0.53, and its standardized coefficient with all covariates entered was 0.21. Self‐blame was no longer associated with ideation once self‐disgust and societal disgust were entered. In a clinical sample from the same report, which did not measure disgust, self‐blame but not other‐blame was associated with ideation.^21^ In a cross‐sectional process model that controlled for depressive symptoms, a statistical indirect association was observed from posttraumatic stress disorder (PTSD) symptoms to suicide risk specifically through the disgusting‐self facet and not through the disgusting‐ways facet.^40^ The specificity of that finding bears directly on the mechanism proposed later in this review.

Longitudinal and intensive longitudinal designs, such as daily diaries, mostly converge with the cross‐sectional findings. In a four‐wave model, emotional abuse and suicidal ideation were reciprocally related at the within‐person level, with self‐disgust mediating both directions.^42^ The four‐wave study of fear of negative evaluation described above also found a one‐way path from that fear to suicidal ideation through self‐disgust.^30^ Not every prospective test is positive. In a three‐wave study of undergraduates (a relatively low‐risk sample), disgust with self carried a statistical indirect association between insomnia symptoms and suicidal ideation in the cross‐sectional analysis at baseline, but not in the longitudinal analysis over the following 2 months. Only disgust with others and with the world mediated the prospective link.^20^ A 7‐day diary study found that, on days of higher perceived stress, students reported more self‐disgust, and more self‐disgust went with more suicidal ideation on the same day.^43^ This indirect association held only at the within‐person level, and the corresponding between‐person effect was non‐significant in a sample of 61 students. Within that limit, the level‐of‐analysis difference points to self‐disgust acting as a daily state and not merely as a trait risk marker.

Cross‐sectional studies add convergent evidence. Self‐disgust was associated with suicidal behavior, alongside PTSD and impulsivity, in young women.^41^ It was one of three variables that carried statistical indirect associations between psychological distress and suicidal ideation in medical students.^22^ In adults recruited online, self‐disgust was associated with the severity of suicidal ideation, which the authors labeled suicide risk, in both sexual‐minority and heterosexual participants, more strongly in the former.^27^ In exploratory analyses from the same study, greater tolerance of shame was associated with lower risk only among sexual‐minority adults with high self‐disgust. Aggregate social‐media data point the same way. Words expressing self‐disgust co‐occurred more strongly with explicit suicidal‐intent words in night‐time than in daytime posts,^44^ although the co‐occurrence of words is not an association within individuals.

The qualitative evidence comes closest to the proposed mechanism. In a grounded‐theory study of men who had attempted suicide, self‐disgust was an important emotion, interwoven with worthlessness and enduring distress, and four of the nine men placed it at the center of their attempt. Its distancing and repellent qualities, together with the fear that one's disgustingness would be exposed, worsened distress and raised suicide risk, and self‐disgust was more pervasive among men with histories of multiple childhood adversities.^3^ Participants described visceral revulsion at the self and efforts to distance themselves from, conceal, cleanse, or change the parts they found disgusting, although they often struggled to separate self‐disgust from shame and self‐hatred. This is the phenomenology that the mechanistic account set out below tries to explain.

## DEVELOPMENTAL AND TRAUMA PATHWAYS

A recurring finding in mediation models is that self‐disgust statistically links retrospectively reported early adversity to self‐harm. This fits evidence that childhood trauma is a risk factor for self‐disgust,^11^ and it fits accounts in which adversity leaves a person feeling disgusting or polluted.^3^ Childhood emotional neglect and sexual abuse are statistically linked to NSSI through self‐disgust.^25^, ^33^ In adults who had recently harmed themselves, adverse childhood experiences were statistically linked to self‐harm through both shame and self‐disgust, although both indirect associations were small.^35^ Adverse childhood experiences are also linked to NSSI through the closely related construct of self‐hate,^46^ which we treat as adjacent rather than identical because it is indexed by a distinct scale. In the suicidality literature, emotional abuse predicted later ideation through self‐disgust, and ideation also predicted later abuse through self‐disgust.^42^ The role of self‐disgust is not confined to self‐harm, because in a cross‐sectional study a statistical indirect association was observed from childhood adversity to positive and negative symptoms of psychosis through self‐disgust.^47^ This suggests that self‐disgust may be a transdiagnostic consequence of adversity that self‐harm research has good reason to track. Trauma‐related distress shows a similar pattern. PTSD symptoms are statistically linked to suicide risk through self‐disgust.^40^ Among combat veterans, betrayal‐based experiences of moral injury, meaning events that violate deeply held moral beliefs, relate to posttraumatic stress symptoms through pathways that include self‐disgust.^48^ The temporal evidence is weaker. In an ecological momentary assessment study, with repeated ratings in daily life, that included people who had experienced sexual assault, self‐disgust and PTSD symptoms rose and fell together over a week, but neither predicted later levels of the other.^49^

Findings in borderline personality disorder are included here only to illustrate how self‐harm and self‐directed disgust co‐occur. Whether measured by self‐report, by taste perception, or by responses to one's own face, disgust is elevated in borderline personality disorder, particularly when it is directed at the self and the body.^24^, ^31^, ^50^, ^51^ Among people with the disorder, self‐disgust is associated with alexithymia, a difficulty in identifying and describing one's own emotions, and with emotion dysregulation.^52^ Self‐disgust is among the internalized symptoms of the disorder that can be distinguished from its externalized, dysregulated behaviors.^53^ We do not address personality functioning or diagnostic debates, which belong to a different literature. The narrower point is that a disorder defined partly by self‐harm is also characterized by heightened self‐ and body‐directed disgust. This co‐occurrence is consistent with the mechanism proposed next, but it does not discriminate between accounts, because shame is also heightened in the disorder.^24^

## WHY THE BODY? THE CONTAMINATION‐AND‐ELIMINATION HYPOTHESIS

The review's central proposal is that self‐disgust, unlike shame or self‐criticism, carries an action tendency that specifically implicates the body, and that this tendency offers a candidate account of why self‐harm takes the bodily forms it does. As noted earlier, disgust functions to keep contaminants out of the body and to stop them from spreading within it.^9^, ^10^ Directed at the self, it is accompanied by efforts to cleanse or suppress disgusting parts of the self and by withdrawal from others.^15^ Disgust normally prompts distance from its object, but no one can move away from their own body. When the contaminated object is the self or the body itself, these tendencies therefore have nowhere external to go, and, according to this account, two behavioral expressions follow. The first is cleansing. Cutting, burning, or otherwise attacking the skin can be read as an attempt to purge or remove the disgusting part. The self‐punishment account predicts the same behavior for a different reason,^2^ so the two can be separated only by asking about motives, including cleansing. Widely used functional measures make this difficult. The Inventory of Statements About Self‐injury assesses 13 functions, including self‐punishment, but none of them concerns cleansing or purging,^54^ so cleansing motives may be under‐represented in quantitative data. Adding cleansing‐motive items to such measures would make the comparison between cleansing and punishment motives testable. The finding that scar‐related shame was associated both with self‐disgust and with self‐punishing, intrapersonal functions of NSSI^32^ fits both accounts. The second is elimination. At the limit, when the contaminated object is the whole self and cannot be cleansed, the disgust action tendency points toward removing the self entirely. On this hypothesis, some people may experience part of their suicidal motivation as the elimination of a contaminated self rather than only as escape from pain. This is a proposed psychological account of one possible motive, not a characterization of suicide in general.

The account builds on earlier suggestions. Clarke et al.^15^ proposed that self‐disgust may drive extreme efforts to cleanse or get rid of the disgusting self, and the qualitative studies they reviewed describe a wish to cut away or wash off the disgusting part. Conceptual work on disgust with life had already linked disgust with oneself to severe suicidal ideation and behavior,^16^ and a review in Chinese proposed that disgust may lead to attacks on oneself and to suicide.^17^ A psychodynamic chapter describes expulsion and attack as defenses linked to self‐disgust, although it focuses on their outward expression in prejudice and violence.^55^ A qualitative study also put forward the hypothesis that self‐disgust may contribute to suicide as an extreme attempt to get rid of a self felt to be contaminated.^3^ The present account extends these suggestions to the bodily forms of NSSI, specifies their scope, and sets out how they could be tested.

Research on mental contamination describes a closely related phenomenon. Mental contamination refers to feelings of contamination that arise without physical contact with a contaminant.^56^ In one study, 60% of women who had been sexually assaulted reported such feelings, and deliberately recalling the assault provoked stronger feelings of dirtiness and a stronger urge to wash than recalling a pleasant memory.^57^ Imagining a non‐consensual kiss produces the same feelings in the laboratory,^58^ and in trauma‐exposed students, self‐disgust was associated with more severe contamination concerns.^59^ In this literature, the behavioral response to internally generated contamination is washing or rinsing, a form of cleansing that does not injure the body. If self‐disgust drives cleansing, the account must therefore explain why it sometimes leads to self‐injury and not only to washing. Candidate moderators include whether the contamination is felt to lie inside the body rather than on its surface, a history of unwanted sexual contact, which predicted rinsing in the laboratory paradigm,^56^ and low disgust toward blood and injury.

The account has a limited scope. The cleansing expression applies most naturally to self‐injury that damages the skin, such as cutting, burning, or picking. It applies less well to self‐poisoning, which requires swallowing a noxious substance, the very act that disgust normally prevents. For self‐harm without suicidal intent, the account therefore predicts a gradient across methods, with self‐disgust more closely tied to cutting and burning than to self‐poisoning. For suicidal acts, the two expressions may point in different directions. The elimination reading predicts an association between self‐disgust and suicidal behavior whatever the method, whereas the cleansing reading predicts a gradient, so studies of suicide attempts would need to test the two predictions separately. A second difficulty is that cutting brings a person into contact with blood and wounds, which are themselves strong elicitors of disgust, and the instinct to avoid pain and injury is one of the natural barriers against self‐injury.^5^ The account therefore implies that people who cut combine high self‐disgust with low disgust toward such stimuli. This prediction has not yet been tested in samples selected for self‐harm. The closest evidence comes from inpatient women with borderline personality disorder, a sample defined by diagnosis rather than by self‐harm. In this group, self‐disgust was higher than in healthy controls, whereas overall disgust sensitivity was lower, as were animal‐reminder disgust, which covers reactions to injury and death, and contamination disgust, but the differences in disgust sensitivity were small, with *η* ^2^ between 0.03 and 0.05.^52^

Three lines of evidence are consistent with this account, although none of them is decisive. First, the finding that PTSD symptoms relate to suicide risk through the disgusting‐self facet and not the disgusting‐ways facet^40^ fits the elimination reading, because it is the self as object, and not merely disapproved behavior, that carries the risk. That result is cross‐sectional, was not a test of the present hypothesis, and fits accounts based on self‐hatred equally well. Second, the body‐image pathway ties the emotion to the body, because cross‐sectional data show a statistical indirect association from body‐image disturbance to suicidal thoughts and behaviors through self‐disgust^39^ and, longitudinally, self‐disgust mediates the path from body‐image concerns to NSSI.^29^ Concerns about fatness or shortness are aesthetic rather than contamination‐based appraisals, however, so this pathway ties self‐disgust to the body without showing that contamination is involved. Third, outside the self‐harm literature, self‐disgust often concerns the person and the body, not only behavior. In patients with mental disorders, self‐disgust about one's physical appearance and personality was more pronounced than self‐disgust about one's behavior.^11^ In a cross‐sectional survey, physical self‐disgust was lower among people with limb amputations who used a prosthesis more often.^18^ Appearance‐directed disgust can be reduced by affirming one's own kindness in non‐clinical samples.^60^ None of these three studies involves self‐harm, so they suggest that the emotion often targets the physical self and can change, not that the proposed mechanism operates in self‐harm.

The evidence for the hypothesis has clear limits. As noted above, the only induction study of self‐disgust and self‐harm urges that we are aware of did not find a significant whole‐sample association between induced disgust and urge change on its analog measureThe evidence for this hypothesis remains limited. As previously noted, the sole induction study examiningself‐disgust and self‐harm urges did not yield asignificant whole‐sample association between induced disgust and changes in urges usingits analogue measure.^31^ The qualitative evidence, though vivid, is retrospective and comes from a small sample whose interviews focused on self‐disgust.^3^ Where the link from self‐disgust to NSSI has been broken down, the indirect paths have run through general processes, emotion regulation difficulties in one cross‐sectional study and depression and psychache in a two‐wave study,^33^, ^37^ which is closer to the general emotion‐regulation account than to a disgust‐specific one. Evidence on suicidal behavior, as distinct from ideation, is cross‐sectional or retrospective, and no study has asked whether self‐disgust distinguishes people who attempt suicide from those who only think about it. To our knowledge, no study has manipulated self‐disgust and then shown a causal downstream effect on self‐harm. The generalizability of the evidence is limited as well. Chinese‐language databases were not searched, so Chinese‐language studies, supportive or null, may be missing, and this matters because most of the longitudinal evidence comes from China. Cultural context may also matter. Self‐disgust measures have been used in Chinese, South Korean, and Japanese samples,^14^, ^41^, ^61^ but none of the studies reviewed here compared the construct or its links to self‐harm across cultural settings. Whether they differ therefore remains an open question. Sex and gender have received little attention as well. Among the 11 studies whose full texts we examined, only one tested gender as a moderator, and the pattern of associations did not differ by gender.^1^ In the others, sex or gender was not tested as a moderator, and some samples included only women or only men. Samples are weighted toward students and toward adolescents in a small number of countries, several key results are cross‐sectional or hold only at the within‐person level, and measures vary in how much bodily content they include. More fundamentally, the main instruments used in these studies, the Self‐Disgust Scale and its revised version, the Questionnaire for the Assessment of Self‐Disgust, and disgust‐with‐life scales, capture revulsion at the self, the body, or behavior, and none of them has a separate contamination subscale. They do not separate contamination appraisals from aesthetic or evaluative dislike. The reviewed studies therefore support the claim that self‐disgust is associated with self‐harm much better than the claim that contamination‐based self‐disgust explains its bodily form. The contamination‐and‐elimination account is therefore advanced as a falsifiable framework, and the research agenda in the next section specifies what would support or refute it.

The account must also be set against rival explanations for why the body is targeted, explanations that it does not yet displace. NSSI may regulate affect by bringing relief from acute negative states, may serve self‐punishment, or may counter dissociation.^2^ None of these functions requires disgust. In survivors of childhood sexual abuse, trauma‐related sadness and fear, as well as disgust, were indirectly related to PTSD severity through self‐harm and derealization, which the authors read as strategies for regulating these emotions.^45^ This argues against a role specific to disgust. Alternatively, the body may be targeted simply because self‐injury is fast and always accessible, an explanation that requires no special emotion at all.^4^ The association may also run in the other direction. Scars and the stigma of self‐injury may themselves make the body feel disgusting, and two of the longitudinal studies found reciprocal paths.^29^, ^30^ What the account adds is a link between the emotion and the form of the behavior. Emotion‐regulation accounts explain why self‐harm brings relief but not why it targets the body, self‐punishment accounts predict harm as deserved suffering, and shame and self‐criticism predict hiding and correction. The cleansing reading instead predicts removal motives, a closer link with physical than with behavioral self‐disgust, and a gradient across methods. The contamination‐and‐elimination account earns its place only if it makes predictions these rivals do not. For example, disgust directed at the body should predict attacks on the body over and above shame, pain sensitivity, and self‐critical cognition, whereas disgust at one's character or behavior should predict withdrawal more than bodily harm. Eating disorders are a sharp boundary case. Self‐disgust scores are among the highest in these disorders,^11^ which involve harm directed at the body. In young adults, eating‐disorder symptoms and body dissatisfaction went with more suicidal ideation only when disgust with the self and the world was high.^19^ Eating disorders therefore offer a test case. If the mechanism holds, body‐directed disgust should also predict self‐harm in these disorders. Whether self‐disgust predicts self‐harm over and above general disgust proneness has, to our knowledge, been examined only cross‐sectionally and only for lifetime suicidal ideation and behavior,^38^ and it remains open for NSSI.

## IMPLICATIONS AND A RESEARCH AGENDA

The implications that follow are hypotheses for clinical research. Their value for predicting or preventing later self‐harm and suicidal behavior has not been established. For assessment, self‐disgust appears to be associated with suicidality partly independently of depression,^40^ of two constructs of the interpersonal theory of suicide,^21^ and of body‐image disturbance.^39^ This suggests that brief self‐disgust measures may add information that mood and belongingness measures miss, particularly where the body is implicated. That increment must be read with one caution built into the construct's measurement. The instruments used, chiefly the Self‐Disgust Scale and its revised version, ask about both the physical and the behavioral self,^23^ and the disgust‐with‐life scale also covers disgust with others and with the world.^20^ The bodily content of self‐disgust items can inflate their associations with body image and with body‐directed self‐harm, and with them any indirect effect that runs from body image through self‐disgust. The same caution applies to depression measures, whose items can share content with self‐disgust items, and the pooled correlation of about 0.52 between the two^28^ shows how large the overlap can be. The strongest claims are therefore those that adjust for the nearest competing construct. The value of self‐disgust in screening remains a proposal to be tested prospectively and not a claim of predictive superiority.

For intervention, two different implications follow. Approaches that reduce global negative self‐evaluation are plausible first candidates, because affirming one's own kindness reduced appearance‐directed disgust in non‐clinical samples.^60^ Compassion‐focused therapy, which aims to replace hostile and disgust‐based self‐talk with kind and supportive self‐talk, has also been proposed in this context.^62^ The contamination logic suggests, however, that disgust may respond to techniques different from those that target sadness or self‐critical thought. Such techniques would target the contamination appraisal itself, for example by examining the belief that the body or the self is contaminated, or by the graded reduction of avoidance and cleansing behaviors. Reassurance that the body is clean is not the aim, because it could strengthen the belief that contamination is the problem to be solved. To our knowledge, this avenue has not been tested for self‐harm. Dialectical behavior therapy, which reduces self‐directed violence,^63^ includes the skill of opposite action, which has been piloted for maladaptive shame in five women with borderline personality disorder.^64^ To our knowledge, neither this skill nor other components of the therapy have been studied for self‐disgust, and opposite action is a natural candidate for such a test. Because self‐disgust can operate in part through depression,^37^ trials should test sequencing and mediation rather than presume them.

The hypothesis yields falsifiable predictions. Because current measures do not separate contamination from other forms of revulsion, studies testing these predictions should measure contamination appraisals and cleansing urges directly. Experimental induction or reduction of self‐disgust should change self‐harm urges, and ideally behavior, more than a matched induction or reduction of shame, and over and above changes in mood. Induction paradigms from research on mental contamination^58^ could be adapted for such experiments, with appropriate safeguards. Physical self‐disgust, measured for example with the physical subscale of the revised Self‐Disgust Scale,^18^ should predict body‐directed self‐harm better than behavioral self‐disgust and general self‐criticism do. In self‐harm without suicidal intent, self‐disgust should be more closely tied to cutting and burning than to self‐poisoning. It should also distinguish people who attempt suicide from those who only think about it. People who report cleansing motives for self‐injury should score higher on self‐disgust than those who report only punishment motives. Interventions that reduce body‐directed disgust should reduce NSSI more than interventions targeting evaluative self‐criticism alone. The account would be refuted if, in adequately powered prospective studies, self‐disgust did not predict NSSI beyond shame and self‐criticism, or if inducing self‐disgust raised self‐harm urges no more than a matched shame induction.

## CONCLUSION

Self‐disgust is a distinguishable, if overlapping, self‐conscious emotion that is associated with NSSI and with suicidal ideation in most studies, and with suicidal behavior in cross‐sectional and retrospective studies. In mediation models—most of which are cross‐sectional—self‐disgust statistically links early adversity and body‐image concerns to self‐harm. In some studies, it adds explanatory value beyond depression and beyond two constructs of the interpersonal theory of suicide. Across disorders, its associations shrink when other negative self‐referential emotions are controlled for. Not every prospective test is positive, and the prospective studies tested different roles with different measures and intervals. The supportive longitudinal evidence comes from at most five independent samples, all in China, and the boundaries of self‐disgust with shame and self‐criticism remain contested. The distinctive contribution of self‐disgust to understanding self‐harm is a hypothesis, not yet tested experimentally, that the contamination‐and‐elimination logic of disgust explains why distress is enacted on the body. On this hypothesis, cutting may in some cases be an attempt to cleanse or to remove the disgusting part, and part of some people's suicidal motivation may be the wish to eliminate a self experienced as contaminated. Read this way, self‐disgust is an embodied emotion, one felt in and directed at the body, distinguishable from shame and low self‐esteem, whose action tendency may match the phenomenology of self‐harm. Whether that match reflects an underlying mechanism is now an empirical question with aclear set of testable hypotheses.

## AUTHOR CONTRIBUTIONS

DJ conceived the review and was responsible for methodology, investigation, visualization, and writing of the original draft. MB contributed to validation and to the review and editing of the manuscript. Both authors read and approved the final manuscript.

## CONFLICT OF INTEREST STATEMENT

The authors declare no conflicts of interest.

## ETHICS APPROVAL STATEMENT

Not applicable. This narrative review did not involve human participants or animals.

## PATIENT CONSENT STATEMENT

N/A.

## CLINICAL TRIAL REGISTRATION

N/A.

## Supporting information

Supporting File 1

## Data Availability

Data sharing is not applicable to this article as no new data were created or analyzed in this study.
