# Supplementary material for: Self‐disgust and the body as a target in self‐harm and suicidal behavior: A narrative review
Source: PCN Rep. 2026 Nov 10;5(4):e70443. doi: 10.1002/pcn5.70443 (PMC13749618; doi:10.1002/pcn5.70443)
Supplement: Supplementary file 1 [file PCN5-5-e70443-s001.docx]

# Supporting Information

Self-disgust and the body as a target in self-harm and suicidal behaviour: a narrative review

Dorin Jelaga and Mihaela Belous

# Supporting Information S1. Literature search

# Databases and timing

The searches were run in September 2026 in PubMed, Europe PMC, and Crossref. Supplementary queries in OpenAlex and Semantic Scholar did not complete because of service errors and contributed no records. The search strings differed between databases. Each PubMed and Europe PMC string is given below with the number of records it returned when it was run. For Crossref, the queries are given, but the numbers of results were not recorded.

# PubMed

| **String** | **Records returned** |
| --- | --- |
| ("self-disgust" OR "self disgust") AND (self-harm OR self-injury OR suicid*) | 31 |
| "self-disgust" AND (self-harm OR self-injury OR "self-injurious" OR nonsuicidal OR suicid* OR borderline) | 39 |
| "self-disgust" AND (scale OR validation OR measure OR "self-conscious" OR shame OR distinct OR schema) (construct and measurement literature) | 78 |
| ("self-disgust"[tiab] OR "self disgust"[tiab] OR "disgust with self"[tiab] OR "self-directed disgust"[tiab] OR "disgusting self"[tiab] OR "self-loathing"[tiab]) AND (suicid*[tiab] OR "self-harm"[tiab] OR "self harm"[tiab] OR "self-injur*"[tiab] OR "self injur*"[tiab] OR NSSI[tiab] OR "self-mutilat*"[tiab] OR "self-cutting"[tiab]) | 41 |
| The first string combined with (review[Title] OR review[Publication Type] OR systematic[Title] OR meta-analysis[Publication Type]) (check for existing reviews) | 0 |

# Europe PMC

| **String** | **Records returned** |
| --- | --- |
| (ABSTRACT:"self-disgust" OR ABSTRACT:"self disgust" OR ABSTRACT:"self-directed disgust" OR ABSTRACT:"disgust with life" OR ABSTRACT:"disgust with oneself" OR ABSTRACT:"self-loathing") AND (ABSTRACT:suicid* OR ABSTRACT:"self-harm" OR ABSTRACT:"self-injur*" OR ABSTRACT:NSSI OR ABSTRACT:"self harm" OR ABSTRACT:"self-mutilation") | 45 |
| (TITLE_ABS:"self-disgust" OR TITLE_ABS:"self disgust") AND (TITLE_ABS:suicid* OR TITLE_ABS:"self-harm" OR TITLE_ABS:"self-injury" OR TITLE_ABS:NSSI) AND (TITLE:review OR TITLE:systematic OR TITLE:"meta-analysis" OR TITLE:scoping) (check for existing reviews) | 1 |
| (TITLE_ABS:"self-disgust" OR TITLE_ABS:"self disgust") AND (TITLE_ABS:suicid* OR TITLE_ABS:"self-harm" OR TITLE_ABS:"self-injury" OR TITLE_ABS:NSSI) AND (FIRST_PDATE:[2025-06-01 TO 2026-12-31]) (check for recent records) | 5 |

# Crossref

Six bibliographic queries were run, with 40 results requested for each. The queries were "self-disgust suicide review", "self-disgust self-harm review", "self-disgust suicidal ideation", "self-disgust self-injury", "self-disgust nonsuicidal self-injury", and "disgust with self suicidal".

Results were kept when the title contained "disgust" together with a self-harm or suicide term. The total numbers of results were not recorded.

# Other sources

A few studies were found in the reference lists of related articles.

# Eligibility

Peer-reviewed articles and book chapters were eligible, and unpublished theses were not included. Publications had to be in English or to have an English abstract. Chinese-language databases were not searched. Table 2 lists the studies identified by these searches that tested self-disgust as a construct in relation to a self-harm or suicidality outcome in individuals. Other studies were included when they informed the construct, its measurement, or the proposed mechanism.

# Selection

DJ directed the searches and the data extraction and made all eligibility decisions. MB reviewed the synthesis and the manuscript. Records were screened on title and abstract. Screening and data extraction were not duplicated, so there were no disagreements between reviewers to resolve. Screening decisions were not logged record by record, so the number of records screened after the removal of duplicates cannot be reported and a flow diagram cannot be reconstructed. For the revised Table 2 and Supporting Information S2, each value was checked against the text of its source, the full text where it was accessible and otherwise the published abstract.

# Supporting Information S2. Informal appraisal of the studies in Table 2

The appraisal is informal and is not a formal risk-of-bias assessment. Studies are listed in the order of Table 2 of the main text.

| **Study** | **Sample type** | **Retention** | **Prior level of outcome controlled** | **Multiple-testing control** | **Sex or gender** | **Overlapping samples** | **Source of values** |
| --- | --- | --- | --- | --- | --- | --- | --- |
| Smith et al. (2015) | Students | – | – | NR | NR | NR | Abstract only |
| Abdul-Hamid et al. (2014) | Clinical and healthy volunteers | Single session | – | Not reported for the disgust and urge tests | Women only | Part of a larger project | Full text |
| Bachtelle and Pepper (2015) | Students | – | – | NR | NR | NR | Abstract only |
| Erol and Inozu (2024) | Students | – | – | NR | NR | NR | Abstract only |
| Jiang et al. (2026) | School | – | – | NR | Gender covariate only | None stated | Full text |
| Mohammadpour et al. (2025) | Community, online | – | – | NR | Not examined | None stated | Full text |
| Ying et al. (2025) | Adolescents | NR | Yes (RI-CLPM by design) | NR | NR | Appears to be the same sample as Zhang et al. (2025) | Abstract only |
| Zhang et al. (2025) | Adolescents (secondary school) | NR | Yes (RI-CLPM by design) | NR | NR | Appears to be the same sample as Ying et al. (2025) | Abstract only |
| Shen et al. (2024) | Adolescents | NR | NR | NR | NR | Different sample, same senior author as Ying et al. (2025) and Zhang et al. (2025) | Abstract only |
| Hong et al. (2026) | Adolescents | NR | NR | NR | NR | NR | Abstract only |
| Hamza et al. (2021) | Students | 83% completed all three waves (FIML) | Yes (autoregressive NSSI paths), but self-disgust tested only as moderator | NR | Gender covariate. Model did not differ by gender, χ²(6) = 4.99, p = .54 | Larger ongoing longitudinal project (no overlap stated) | Full text |
| Chu et al. (2015) | Young adults | – | – | NR | NR | NR | Abstract only |
| Schienle et al. (2020) | Community, online | – | – | NR | NR | NR | Abstract only |
| Akram et al. (2022) | Students and community, online | – | – | NR | No sex differences in self-disgust or outcome. Sex as covariate | None stated | Full text (journal pre-proof) |
| Robison et al. (2025) | Community, online | – | – | NR | Not examined | None stated | Full text |
| Brake et al. (2017) | Students (trauma-exposed) | – | – | NR | NR | NR | Abstract only |
| Gang et al. (2026) | Community | – | – | NR | Women only | NR | Abstract only |
| Amraei et al. (2025) | Students | – | – | NR | Not examined | None stated | Full text |
| Mann et al. (2022) | Community, online (MTurk) | – | – | NR | Sex screened as covariate, not retained | Parent online sample of 515 (no overlap with other publications stated) | Full text |
| Gong and Zhou (2025) | School | NR | Yes (RI-CLPM by design) | NR | NR | NR | Abstract only |
| Hom et al. (2019) | Students (relatively low-risk) | 79% completed all waves (listwise deletion) | Yes | NR | Not examined (more men dropped out) | None stated | Full text |
| Wang et al. (2026) | Students | 61 of 68 retained | No (same-day associations) | NR | Covariate in sensitivity analysis only | None stated | Full text |
| Mason et al. (2022) | Clinical and community | – | – | – | Men only | None stated | Full text |

Retention applies to designs with more than one assessment. Prior level of outcome controlled indicates whether an earlier measurement of the self-harm or suicidality outcome was taken into account. Overlapping samples records samples shared with another study in Table 2. For the studies marked Abstract only, the full text was not accessible to the authors, and the values come from the published abstract. FIML = full-information maximum likelihood, NR = not reported in the source used, RI-CLPM = random-intercept cross-lagged panel model, SDS = Self-Disgust Scale, – = not applicable.
